# Optimization of Growth Conditions for Chlorpyrifos-Degrading Bacteria in Farm Soils in Nakuru County, Kenya

**DOI:** 10.1155/2024/1611871

**Published:** 2024-01-25

**Authors:** Miriam Wepukhulu, Peter Wachira, Nderitu Huria, Paul Sifuna, Suliman Essuman, Micah Asamba

**Affiliations:** ^1^School of Biological Sciences, University of Nairobi, P.O. Box 30197-0100, Nairobi, Kenya; ^2^Department of Dryland Agriculture and Natural Resources, Tharaka University, Marimanti, Kenya; ^3^Department of Medical Microbiology, Mount Kenya University, P.O. Box 342-01000, Thika, Kenya; ^4^Department of Biochemistry, Microbiology, and Biotechnology, Kenyatta University, P.O. Box 43844, Nairobi, Kenya

## Abstract

Chlorpyrifos (CP) is a chlorinated organophosphate pesticide. In Kenya, it is commonly used as an acaricide, particularly in dairy farming, leading to soil and water contamination. The study is aimed at isolating bacteria with CP-degrading potential and optimizing their growth conditions, including temperature, pH, and CP concentration. The enrichment culture technique was used, with minimal salt medium (MSM) supplemented with commercial grade CP. A multilevel factorial design was used to investigate the interactions of temperature, pH, and CP concentration. According to the findings, seven bacterial strains with potential to degrade CP were characterized and identified as *Alcaligenes faecalis*, *Bacillus weihenstephanensis*, *Bacillus toyonensis*, *Alcaligenes* sp. strain *SCAU23*, *Pseudomonas* sp. strain *PB845W*, *Brevundimonas diminuta*, and *uncultured bacterium* clone 99. Growth and biodegradation of bacteria differed significantly among the isolates across pH value, temperature, and concentrations (*P* ≤ 0.05). The optimum conditions for growth were pH 7, temperature of 25°C, and 25mg/l chlorpyrifos concentration, while optimum degradation conditions were pH 5, temp 25°C, and CP conc. 25mg/l. The Pearson correlation between optimum growth and degradation showed a weak positive relationship (*R* = 0.1144) for pH and strong positive relationship for temperature and concentration of chlorpyrifos. Other than pH, the study shows that there could be other cofactors facilitating the chlorpyrifos degradation process. The findings show that an efficient consortium, at 25°C and pH 5, can include *Bacillus toyonensis 20SBZ2B* and *Alcaligenes* sp. *SCAU23* as they showed high optical density (OD) values under these conditions. These results indicate the potential for these bacteria to be employed in chlorpyrifos-contaminated ecosystem detoxification efforts upon manipulation of natural growth conditions. The findings of this study offer a potential foundation for future research into the reconstitution of a consortium. Based on the optimum conditions identified, the isolated bacterial strains could be further developed into a consortium to effectively degrade CP in both laboratory and field conditions. Dairy farmers can utilize the isolated strains and the consortia to decontaminate farm soils.

## 1. Introduction

Pesticides are an important part of the agricultural business and are widely utilized as part of pest control strategies in agriculture. Globally, pesticides are utilized in excess of 5.6 billion pounds annually [1]. An ideal pesticide is dangerous exclusively to the creatures it is designed to kill, is biodegradable, and does not pollute the environment [2]. Notably, pesticides applied indiscriminately can harm nontarget organisms and unintentionally reach other ecosystems. As reviewed by Poudel et al., only 0.1% of applied pesticides reach the target pests and 99.9% escape into the environment, where they pose a threat to public health and beneficial biota and contaminate the ecosystem [3].

Chlorpyrifos (CP), a type of organophosphate (OP) acaricide, accounts for 38% of global pesticide use but lacks target specificity, posing risks to nontarget species [4, 5]. Widely used in Kenya for tick control in dairy animals, its extensive application threatens human health and environmental integrity by contaminating air, soil, and water [6–8]. Unrestricted use by farmers can lead to acute diseases, loss of beneficial biota, and ecosystem imbalances, including altered soil microbial communities [9, 10]. In Kenya, many agricultural and pesticide firms lack adequate treatment facilities for organophosphates (OPs), leading to environmental contamination [2, 11, 12]. Various remediation methods have been explored but have limitations in cost, effectiveness, or environmental impact [13–18].

Bioremediation using autochthonous microorganisms is an emerging approach for detoxifying pollutants like chlorpyrifos [19, 20]. While pure cultures have been studied, natural conditions often involve complex microbial consortia, making enriched cultures from contaminated sites more effective [21]. Various factors, including microbial components and physicochemical conditions, influence the rate of chlorpyrifos degradation. However, there is limited knowledge on optimizing these conditions for effective biodegradation [4].

The study is aimed at identifying bacteria in Nakuru County's agricultural soils capable of degrading chlorpyrifos (CP) and at optimizing their growth conditions for effective bioremediation. Specifically, the research focuses on isolating bacterial strains with CP degradation potential and determining their optimal growth conditions in terms of pH, temperature, and CP concentration. This work seeks to advance bioremediation techniques for CP-contaminated environments.

## 2. Materials and Methods

### 2.1. Reagents

Chlorpyrifos of analytical grade (99.4%) was procured from Sigma-Aldrich (USA). The research utilized a mineral salt medium (MSM) consisting of the following components in grams per liter (g/l): Na_2_HPO_4_ at 5.8, KH_2_PO_4_ at 3.0, NaCl at 0.5, NH_4_Cl at 1.0, and MgSO_4_ at 0.25. Concentrated stock solutions of CP (10 g/l) were produced and passed through 0.22 mm syringe filters. The process of achieving medium sterilization involved subjecting it to autoclaving at a temperature of 121°C for a duration of 15 minutes.

### 2.2. Soil Sampling Procedure

Soil samples were collected from Molo, Njoro, and Subukia in Nakuru County, Kenya (latitude: 0° 29′ 59.99^″^ N; longitude: 36° 00′ 0.00^″^ E). Nakuru experiences a warm and temperate climate, with annual rainfall of approximately 762 mm and a 17.5°C temperature. Sampling was carried out in 15 selected dairy farms with a history of repeated chlorpyrifos acaricide application through cattle dips and spray races according to standard operation procedures (SOP Number: FSS0002.00) and European guidelines [22]. All samples were code-named, stored in cool boxes with ice packs, and then transported to Mount Kenya University Research Laboratory. The soil samples were air-dried and sieved.

### 2.3. Determination of Soil Physicochemical Properties

In situ measurements for pH, electrical conductivity, and total dissolved solids were performed at specific sampling points, in compliance with APHA (2005) guidelines. For electrical conductivity, a Jenways 4076 EC meter was used, calibrated across a range of 0 to 200 millisiemens per meter at 25°C. The probe was submerged directly in the water for accurate readings. pH was determined on-site using a Jenways 3071 portable pH meter, and total dissolved solids were quantified with a Jenways 4076 TDS meter. Furthermore, laboratory investigations were performed to evaluate additional soil properties, in conjunction with the aforementioned in situ observations. The parameters encompassed in the analysis were total nitrogen, organic carbon, phosphorus, potassium, calcium, magnesium, manganese, copper, iron, zinc, and sodium. The laboratory analysis was conducted at the National Agricultural Research Laboratories of the Kenya Agricultural and Livestock Research Organization (KALRO), Kabete, Kenya.

#### 2.3.1. Isolation and Purification of the Most Common Microbes

The study collected soil samples from three distinct locations in Nakuru County, Kenya, namely Molo, Njoro, and Subukia. The geographical coordinates of the study sites were latitude of 0° 29′ 59.99^″^ N and longitude of 36° 00′ 0.00^″^ E. The climate in Nakuru is characterized by warm and temperate conditions, with an average annual precipitation of around 762 mm and a temperature of 17.5°C. The study involved the implementation of standardized operating procedures (SOP number: FSS0002.00) and adherence to European guidelines [22] during the sampling process, which was conducted in 15 dairy farms that had a documented history of repeated use of chlorpyrifos acaricides through dips and spray races. The specimens were assigned code names, placed in refrigerated containers with ice packs, and subsequently conveyed to the research laboratory at Mount Kenya University. The soil samples underwent an air-drying process and were subsequently subjected to sieving.

### 2.4. Experimental Design

The relevance and interactions of three independent variables, temperature (25°C, 30°C, and 37°C), pH (values 5, 7, and 9), and CP concentration (25mg/l, 50 mg/l, and 100 mg/l), were investigated using a general multilevel factorial design. A multilevel factorial design allows for some flexibility in the number of levels used for each independent variable. For the purpose of assessing the experimental error, the centre point was duplicated three times. Using a 2^3^-factorial design, all independent variables were combined to create a design matrix.

### 2.5. Biodegradation of Chlorpyrifos by the Selected Isolates

For the biodegradation test, pure cultured isolates in mineral salt liquid medium (MSM) enriched with chlorpyrifos (10 mg/l) were utilized. Pure cultures of isolated strains with an inoculum density of 2.4 × 10^6^ CFU ml^−1^ were cultured on a rotary shaker for 3 days at 150 rpm and 30°C. The experiment was repeated three times, with controls consisting of media without inoculation kept under the same conditions. The growth of bacteria was evaluated turbidometrically at different time intervals by measuring optical density in Spectronic 20 spectrophotometer at 600 nm [23]. One milliliter of sample was taken and added to freshly prepared 0.02% tetrazolium chloride in a boiling tube and the contents of the boiling tube boiled for 5 minutes in a Stuart water bath SWB series. The boiling tubes and contents were incubated for 21 days, and optical density was taken at intervals (OD_480_) and color change observed.

The isolated microbial strains were cultivated on MSM supplemented with chlorpyrifos (10 mg l^−1^) and examined for their ability to grow and degrade CP at different pH values (5, 7, and 9), temperature degrees (25°C, 30°C, and 37°C), and concentrations (50 mg/l, 100 mg/l, and 150 mg/l) to optimize the growth conditions for biodegradation. At all intervals, control flasks containing an equivalent volume of MSM and chlorpyrifos, but no microbial population and MSM with inoculum without CP were cultured. For the purpose of estimating chlorpyrifos degradation, samples were collected and extracted at intervals of 0, 1, 2, 3, 4, and 5 days during the experiment. Seven bacteria code-named MW1, MW2, MW3, MW4, MW5, MW6, and MW7 were selected for characterization based upon their eminent propensity to efficiently degrade CP.

### 2.6. Identification of CP-Degrading Bacterial Strains

Seven bacterial isolates (MW1-MW7) were selected for their ability to biodegrade CP, identified morphologically based on Bergey's Manual and confirmed by 16S rRNA gene analysis. DNA was extracted and amplified using PCR with universal primers, and the resulting amplicons were purified and sequenced (36-40). The sequences were analysed using BLAST to compare them to sequences in the GenBank databases.

### 2.7. Determining the Biodegradability of Chlorpyrifos by Tetrazolium Reduction Assay

The Bhagobaty and Malik method of tetrazolium chloride reduction test was employed with certain alterations to evaluate the biodegradation of chlorpyrifos (CP) by the bacterial isolates [24]. Tetrazolium chloride functions as an artificial electron acceptor that is enzymatically reduced by bacterial dehydrogenase upon initiation of CP degradation by the isolate, leading to the production of the vividly pigmented formazan. The ability of the isolate to degrade CP in a qualitative manner is evidenced by the production of a vividly crimson product. The absence of a discernible alteration in coloration within the control group indicates that the compound CP did not undergo degradation in the MSM that was not inoculated. In a concise and sterile manner, the autoclaved mineral salt media (MSM) was combined with 150 mg/l of CP, which functioned as the exclusive carbon source, within boiling tubes. A recently cultivated culture was introduced into a growth medium and subjected to incubation in a rotary shaker at a temperature of 30°C for a duration of 48 hours. After incubation, 1 ml of the sample was removed and combined with 5 ml of freshly made 0.02% tetrazolium chloride in the test tubes containing the organism. After a 5-minute boiling, the test tubes were incubated at 30°C for 4 hours at ambient conditions. The conversion of colorless tetrazolium chloride into purple Formazan showed positive biodegradation. The biodegradation was quantified using UV-Vis spectrophotometer at optical density (OD) 480 nm at 4-day intervals for a duration of 21 days.

### 2.8. Statistical Analysis

To determine the significance of differences in treatment means, data on bacterial growth and chlorpyrifos biodegradation in MSM were computed using factorial analysis of variance (ANOVA) with Statistical Analysis Software (SAS) 2010. Mean pair-wise comparison was carried out using Tukey's HSD (honestly significant difference) at 5% level. A *P* ≤ 0.05 was considered significant.

## 3. Results

### 3.1. Soil Physicochemical Properties

The soil analysis reveals key physicochemical properties for a farm in Nakuru. Total nitrogen was found to be adequate across samples, ranging from 0.37% to 0.46%. Total organic carbon also fell within adequate levels, with percentages between 3.94% and 4.94%. In terms of essential nutrients measured in meq%, potassium levels were high, varying from 10.0 to 12.8. Calcium and magnesium followed suit, with high levels at 16.0 to 19.0 and 5.78 to 6.78, respectively. Sodium was adequate, ranging from 0.64 to 1.06. The soil pH varied from medium alkaline at 8.18 to near neutral at 6.92 and 6.95. The high nutrient levels suggest a likely clay-loam texture. Electrical conductivity was high at 1.25 mS/cm, indicating good ion exchange capacity but potential salinity concerns (Table 1).

### 3.2. Isolation and Identification of Chlorpyrifos-Degrading Bacteria

Scientific reports have thus far identified only a few species of bacteria capable of degrading chlorpyrifos (CP) and its main metabolite (TCP). By using the enrichment culture technique and MSM media, the current study isolated seven bacterial strains (MW1-MW7) from CP-contaminated soils. The growth of the bacteria in the MSM broth was confirmed through spectrophotometry, and degradation of the CP was confirmed through a positive test of the tetrazolium chloride reduction assay. The tests showed that there was no color change in the control, in which there was no degradation. The small subunit 16S rRNA gene analyses and phylogenetic investigation using BLAST software conferred that the isolates MWI to MW7 were identical to *Alcaligenes faecalis*, *Bacillus weihenstephanensis*, *Bacillus toyonensis*, *Alcaligenes* sp. strain *SCAU23*, *Pseudomonas* sp. strain *PB845W*, *Brevundimonas diminuta*, and *uncultured bacterium* clone 99, respectively, with a similarity of between 90 and 100%. The strains' 16S rRNA nucleotide sequences have been deposited in NCBI GenBank under accession numbers MZ359822.1-MZ359828.1 (Supplemental materials (available here)).

### 3.3. Qualitative Analysis of CP Utilization through Tetrazolium Reduction Assay

**Figure 1:**
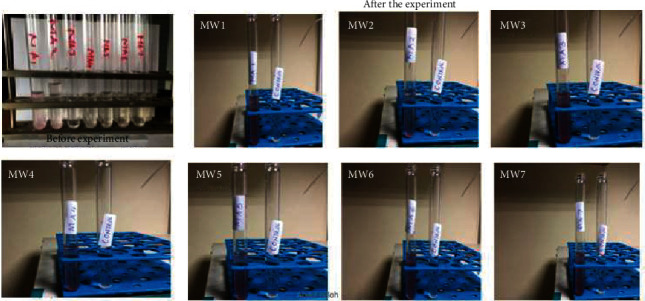
Tetrazolium test results showing color change to purple in all 7 isolates. The image on the top left shows the setup before the experiment. Images MW1 to MW7 show the change in color in the inoculated tubes to purple (due to degradation of CP), while the control remained colorless, indicating no degradation. Key: MW1: *Alcaligenes faecalis UWI9*; MW2: *Bacillus weihenstephanensis FB25M*; MW3: *Bacillus toyonensis 20SBZ2B*; MW4: *Alcaligenes* sp. *SCAU23*; MW5- *Pseudomonas* sp. *P_B845W*; MW6: *uncultured bacterium clone 99*; MW7: *Brevundimonas diminuta CB21*.

The tetrazolium reduction test is aimed at assessing the oxidation of CP by the isolates in the MSM. Once degradation commences, the TTC serves as an electron acceptor that is reduced by the bacteria's dehydrogenase and changed to formazan, which is a highly colored product. Therefore, the presence of purple color is a qualitative measure of degradation. The isolates obtained in the current study were assessed, and color change was reported in all isolates, while there was no color change in the control (Figure 1).

### 3.4. Effect of pH on Bacteria Growth and Biodegradation of Chlorpyrifos

The pH is one of the critical factors that impact bacteria growth and the degradation of xenobiotics. The growth of the isolates was monitored for 21 days at different pH conditions. The effect of pH on the growth of each bacterial isolate was examined by analysing optical density (OD_600_) periodically for a duration of 21 days. Supplementary table S1 gives a summary of data on the effect of pH on bacterial growth in MSM.

**Figure 2:**
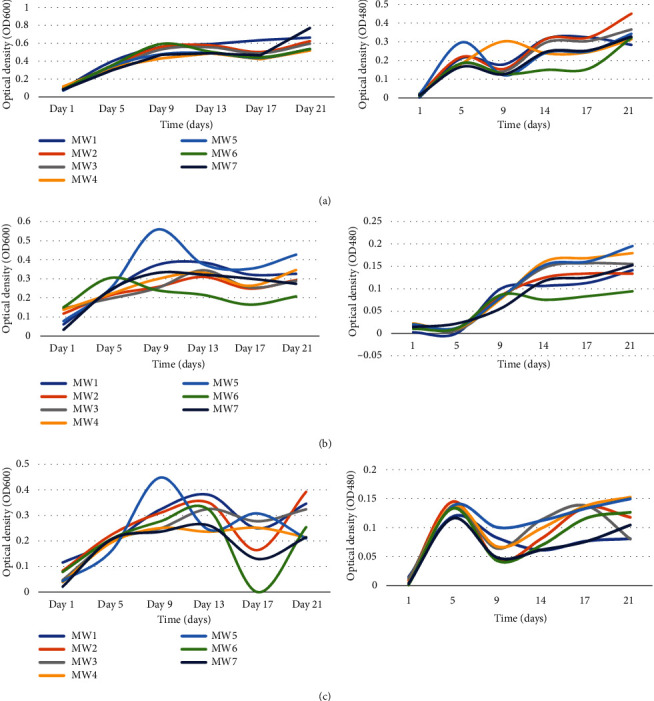
Growth patterns of bacteria isolates (OD_600_) and CP degradation (OD_480_) at pH 5 (a), pH 7 (b), and pH 9 (c). MW1: *Alcaligenes faecalis UWI9*; MW2: *Bacillus weihenstephanensis FB25M*; MW3: *Bacillus toyonensis 20SBZ2B*; MW4: *Alcaligenes* sp. *SCAU23*; MW5: *Pseudomonas* sp. *P_B845W*; MW6: *uncultured bacterium clone 99*; MW7: *Brevundimonas diminuta CB21.* Optical density at 600 nm was used for indirect measurement of bacterial growth, while optical density at 480 nm was to measure the CP degradation as indicated by the intensity of formazan.

The growth patterns and CP-degrading patterns of the different bacterial strains at different pH levels are presented in Figures 2(a)–2(c). It was observed that isolate growth was maximum near the neutral pH conditions, with optimal values for most isolates recorded at pH 7, except MW7 and MW4 which showed highest growth in pH 5. On the other hand, optimum CP degradation was reported at pH 5. Highly basic conditions were characterized with less growth and degradation potential. The analysis of variance (ANOVA) showed that the differences in growth patterns across the three pH levels were significant (*P* < 0.05), with most isolates favouring neutral to slightly acidic conditions. Statistical analysis revealed that optical densities/growth at each pH value differed significantly among the isolates (*P* ≤ 0.05). Besides, a Pearson correlation analysis revealed significant correlation between growth and degradation for all the isolates (*P* < 0.01).

### 3.5. Effect of Temperature on Bacteria Growth and Biodegradation of Chlorpyrifos

**Figure 3:**
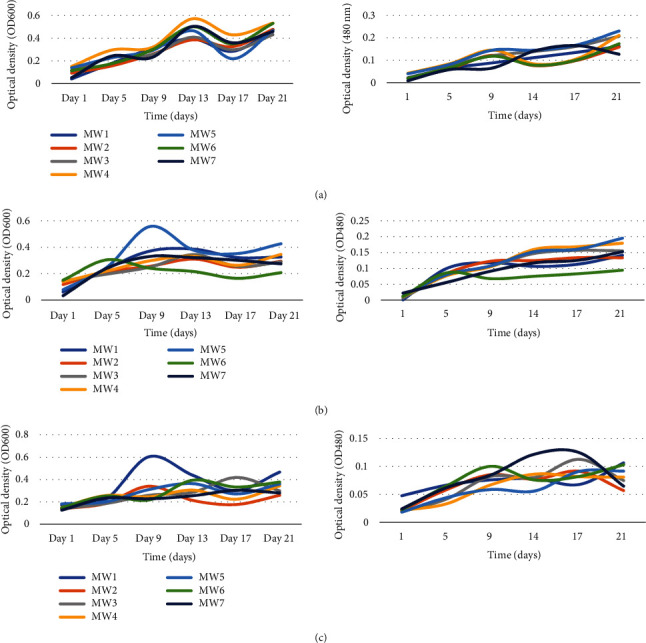
Growth patterns of bacteria isolates (OD_600_) and CP degradation (OD_480_) at 25°C (a), 30°C (b), and 37°C (c). MW1: *Alcaligenes faecalis UWI9*; MW2: *Bacillus weihenstephanensis FB25M*; MW3: *Bacillus toyonensis 20SBZ2B*; MW4: *Alcaligenes* sp. *SCAU23*; MW5: *Pseudomonas* sp. *P_B845W*; MW6: *uncultured bacterium clone 99*; MW7: *Brevundimonas diminuta CB21*. Optical density at 600 nm was used for indirect measurement of bacterial growth, while optical density at 480 nm was used to measure the CP degradation as indicated by the intensity of formazan, a product of tetrazolium breakdown by the bacterial isolates.

The growth of study isolates and CP degradation were investigated at different ranges of incubation temperatures. Bacterial growth and CP degradation were periodically measured across the 21-day period, and the optical densities are summarized in Supplementary table S2. Optical densities for the isolates across temperatures and incubation period were statistically different at *P* ≤ 0.05. The growth differed significantly between inoculated and uninoculated conditions. The growth patterns of the different bacterial strains at different temperature are presented in Figures 3(a)–3(c). The growth of bacterial isolates was determined to be significantly different at different temperatures (one-way ANOVA, *P* < 0.05). The optical densities for both growth and degradation were significantly higher at 25°C compared to 30°C and 37°C. At 25°C, MW5 had the highest growth (OD = 0.533), MW2 at 30°C (OD = 0.426), and MW1 at 37°C. Although the strains showed positive growth and degradation across the three temperatures, the optimum temperature for majority of the strains was 25°C, with the exception of MW2 that had optimum growth at 30°C. Nevertheless, the strains tolerated a wide range of temperature, which is important for in situ bioremediation. Besides, a Pearson correlation analysis revealed significant correlation between growth and degradation for all the isolates (*P* < 0.01). This inference is based on a comprehensive assessment of growth rates and chlorpyrifos degradation, combining graphical and statistical analyses (Supplementary table S2) to identify trends not immediately visible in the graphical data.

Based on the data, MW1 (0.4734 nm), MW3 (0.4304 nm), MW4 (0.46555), MW5 (0.2974 nm), MW6 (0.37665 nm), and MW7 (0.2788 nm) had high ODs at 25°C on day 21 (*P* ≤ 0.05). On the other hand, MW2 (0.47795 nm) showed a high OD at 30°C on day 21 (*P* ≤ 0.05). Statistical comparison of specific ODs among isolates and incubation periods showed statistical significance at *P* ≤ 0.05. The effect of the interaction of main factors on ODs revealed a significant difference in ODs for all isolates at *P* = 0.0001. A comparison between main factors and their interactions revealed that there was a significant difference between ODs for the isolates at incubation intervals (*P* ≤ 0.05). The effect of the interactions of main factors on ODs revealed that there was a significant difference among the ODs for all the isolates at *P* = 0.0001. Besides, a Pearson correlation analysis revealed significant correlation between growth and degradation for all the isolates (*P* < 0.01).

### 3.6. Effect of CP Concentration on Bacterial Growth and Biodegradation of Chlorpyrifos

**Figure 4:**
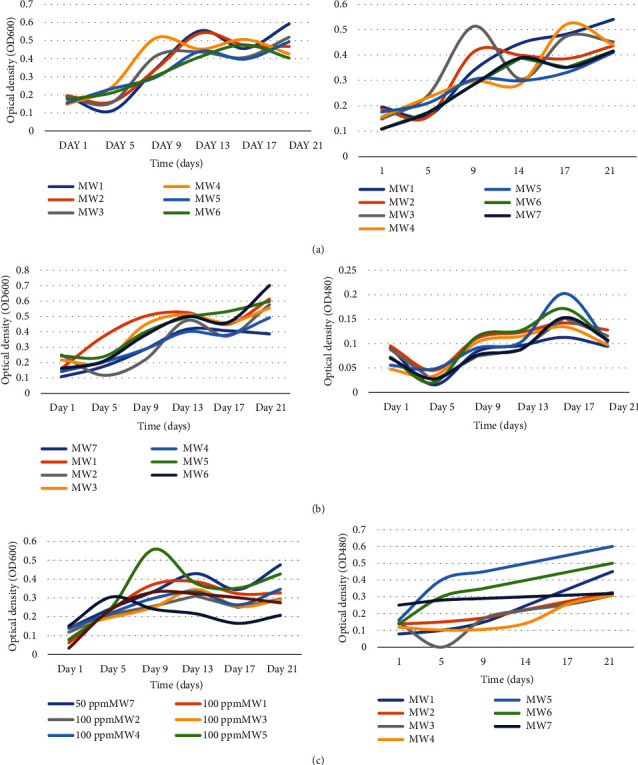
Growth patterns of bacteria isolates (OD_600_) and CP degradation (OD_480_) at 25 mg/l (a), 50 mg/l (b), and 100 mg/l (c). The bacteria isolates are as follows: MW1: *Alcaligenes faecalis UWI9*; MW2: *Bacillus weihenstephanensis FB25M*; MW3: *Bacillus toyonensis 20SBZ2B*; MW4: *Alcaligenes* sp. *SCAU23*; MW5: *Pseudomonas* sp. *P_B845W*; MW6: *uncultured bacterium clone 99*; MW7: *Brevundimonas diminuta CB21*. Optical density at 600 nm was used for indirect measurement of bacterial growth, while optical density at 480 nm was used to measure the CP degradation as indicated by the intensity of formazan, a product of tetrazolium breakdown by the bacterial isolates.

The relationship of specific concentrations with each isolate was analysed, and the interaction between the main factors was determined by assessing optical densities at different intervals across the 21-day incubation period (Supplementary table S3). The growth patterns of the isolates at different CP concentration are presented in Figures 4(a)–4(c). MW1 (0.38675 nm), MW3 (0.5179 nm), MW4 (0.4254 nm), MW5 (0.4926 nm), MW6 (0.403 nm), and MW7 (0.3062 nm) had high ODs at 25 mg/l on day 21 (*P* ≤ 0.05). On the other hand, MW2 (0.46665 nm) showed a high OD at 50 mg/l (*P* ≤ 0.05). Statistical comparison of specific ODs among isolates and incubation periods showed statistical significance at *P* ≤ 0.05. The effect of the interaction of main factors on ODs revealed a significant difference in ODs for all isolates at *P* = 0.0001. A comparison between the main factors and their interactions revealed that there was a significant difference between ODs for the isolates at incubation intervals (*P* ≤ 0.05). The effect of the interactions of main factors on ODs revealed that there was a significant difference among the ODs for all the isolates at *P* = 0.0001. Nonetheless, generally, there was significant growth and degradation across all the concentrations, underscoring the fact that the isolates can adapt to different CP concentrations, although the growth rate and degradation ability decreases slightly at higher concentrations.

From the information in Table 2, the optimum parameters for an effective consortium to degrade CP are a pH of 5 and a temperature of 25°C. The consortium should be diverse to ensure it effectively degrades the pesticide in a broad range of environments. Based on the data provided, a possible consortium could include *Bacillus toyonensis 20SBZ2B* (MW3) and *Alcaligenes* sp. *SCAU23* (MW4) at 25°C and pH 5, as both the strains have shown high OD values under these conditions (Table 2). For a consortium at 30°C, *Bacillus weihenstephanensis FB25M* (MW2) could be included in addition to the strains mentioned above. It is important to note that the growth of the isolates was generally positive across all the three concentrations (25, 50, and 100 mg/l), which suggests that a consortium of the bacteria can be made for all the three concentrations tested. Besides, most bacterial strains display a high degree of congruence between optimum conditions for growth and CP degradation. However, deviations occur primarily in pH and concentration. Specifically, four strains (MW1, MW3, MW5, and MW6) shift to a lower pH for degradation. Only MW2 changes its optimum temperature for degradation. Three strains (MW5, MW6, and MW7) prefer higher concentrations for degradation, likely indicating robustness in coping with substrate abundance. These shifts imply metabolic adaptations and perhaps varying enzymatic activities across different environmental conditions.

### 3.7. Correlation between Bacterial Growth and Chlorpyrifos Degradation

**Figure 5:**
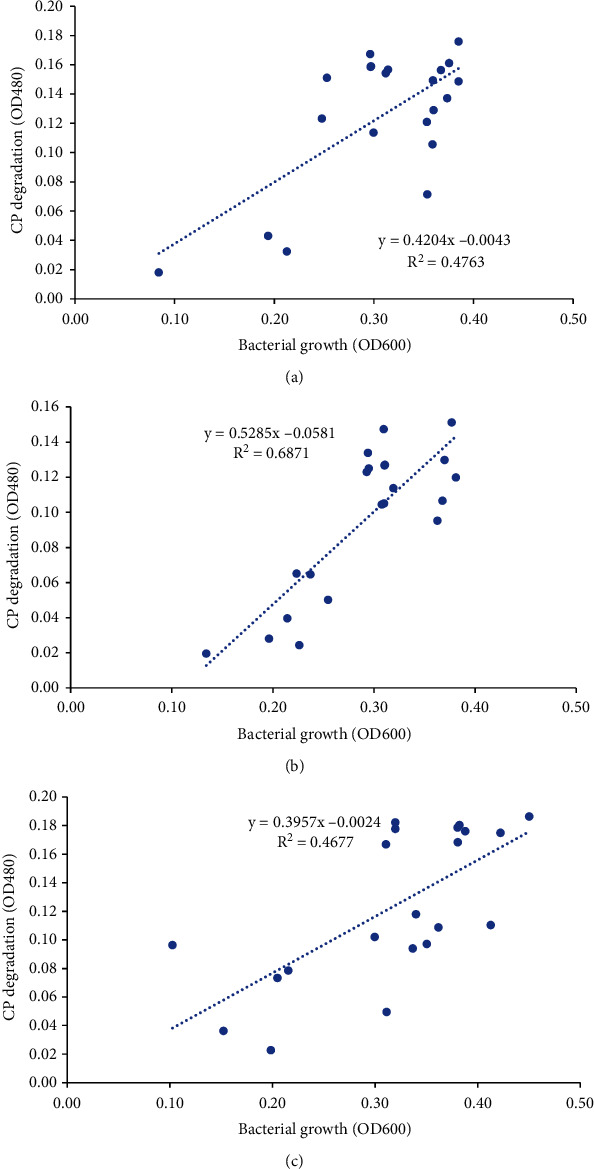
(a) Correlation between bacterial growth and CP degradation at optimum pH 5. (b) Correlation between bacterial growth and CP degradation at optimum temperature 25°C. (c) Correlation between bacterial growth and CP degradation at optimum concentration 25 mg/l.

The Pearson correlation analysis was carried out to determine the correlation between bacterial growth and degradation of chlorpyrifos at the optimum pH, temperature, and concentration. In all the three parameters, a weak, positive correlation was found between growth and degradation (*P* < 0.05) (Figure 5).

## 4. Discussion

The study isolated bacteria with CP-degrading potential and optimized their growth and degradation conditions. The study was necessary as the excessive use of OP pesticides has caused harmful ecological impacts. The biodegradation of chlorpyrifos by microorganisms is a promising solution to reduce its negative impact [1, 4]. This study was among the first to identify the optimum temperature, pH, and substrate concentration required for the *in situ* biodegradation of chlorpyrifos, which could potentially facilitate the development of an effective bioremediation consortium for chlorpyrifos-contaminated environments.

Seven bacterial strains, namely, *Alcaligenes faecalis*, *Bacillus weihenstephanensis*, *Bacillus toyonensis*, *Alcaligenes* sp. *strain SCAU23*, *Pseudomonas* sp. *strain PB845W*, *Brevundimonas diminuta*, and *uncultured bacterium clone 99*, were successfully isolated and characterized from contaminated soils in Nakuru County. These soils have been subjected to repeated and sustained exposure to chlorpyrifos (CP) and other pesticides, resulting in their contamination. The study's findings align with previous research, affirming that bacteria can adapt and thrive in contaminated environments [18, 20, 25]. Over time, these bacterial strains have developed resistance mechanisms in response to repeated exposure to xenobiotic compounds, enabling them to efficiently decompose and remediate contaminated environments [26–28]. Importantly, the growth response of the isolated bacteria in minimal salt medium (MSM) supplemented with chlorpyrifos revealed that they exclusively utilized CP as their carbon source, with qualitative confirmation of their biodegradation potential using tetrazolium chloride. These results emphasize the potential of these bacterial strains for effective bioremediation of chlorpyrifos-contaminated environments.

Since CP has been used for a long time, several researchers have isolated microbes that degrade the pesticide [20, 24, 29–39]. However, only a few studies have tried to optimize the growth conditions for the degrading bacteria. This study stands out as one of the first to rigorously assess the optimum conditions for indigenous bacteria capable of degrading CP. By addressing this crucial aspect, the study makes a significant contribution to the field of bioremediation. Moreover, the emphasis on utilizing indigenous species underscores the importance of ecofriendly approaches to tackle pesticide contamination, minimizing potential disruptions to microflora [29, 31]. These findings carry profound implications, potentially unlocking targeted and efficient bioremediation strategies for chlorpyrifos-contaminated environments, a critical step towards mitigating the detrimental impacts of this hazardous pesticide.

The optimal pH for the growth of majority of the bacteria species was found to be pH 7, with the exception of *Alcaligenes* sp. *Strain SCAU23* and *uncultured bacterium clone 99*, which showed optimum growth at pH 5. These pH levels resulted in the highest optical densities, indicating the most favorable conditions for bacterial growth. Previous studies have determined the optimal growth pH for these bacteria to be pH 7.5 for *Pseudomonas* sp. [40], pH 7.0 for *Bacillus* sp. and *Brevundimonas diminuta CB21* [40], pH 5.8-7.0 for *Alcaligenes faecalis* [41], pH 6.5 for *Bacillus toyonensis* [41], and pH 5.4-7.0 for *Bacillus weihenstephanensis* [42]. On the other hand, the optimum pH for degradation was shown to be pH 5, with the exception of only *Alcaligenes faecalis*, which showed optimum degradation at pH 7. It is important to note that addition of CP into the growth medium may have forced the bacteria to adapt to new conditions, hence accounting for the variations in optimum pH for growth and for degradation. The prevalence of the pH 5 for degradation of CP suggests that most of the isolates prefer acidic conditions as favorable for degradation [43]. However, Farhan et al. [44] isolated *Bacillus* sp. *Ct3* that degraded 88% of CP within 8 days under alkaline conditions. Therefore, optimizing the pH level is important for successful bacterial growth in chlorpyrifos environments. It is important to recognize that the current study's evaluation of three pH levels (5, 7, and 9) aimed to provide a comprehensive understanding of bacterial growth and CO degradation in varied environmental conditions. The levels used in this study had extremes beyond what previous studies had noted to be optimum (between pH 5 and 8), with the aim of determining the resilience and adaptability of the strains in harsh environments.

These findings corroborate previous studies that have demonstrated the critical role of pH in bioremediation [45]. The results align with previous studies demonstrating that neutral to slightly acidic pH is crucial for maximum chlorpyrifos biodegradation [19, 25, 46–48]. These results align with Vidali's findings (2001), indicating that microbial strains in polluted environments often exhibit favorable growth between pH 5 and 8. Additionally, the study's optimal growth pH for *Pseudomonas* sp. and *Bacillus weihenstephanensis* (pH 7.0 and pH 5.0 to 7.0, respectively) further validate the findings reported by Singh et al. [47]. The finding that *Brevundimonas diminuta CB21* demonstrated the highest growth at all pH levels among the bacterial species tested is a significant result. It suggests that this bacterial species has the potential to be an effective candidate for bioremediation of chlorpyrifos-contaminated environments [31]. The use of such bacteria could potentially mitigate the adverse effects of chlorpyrifos pollution on the environment and human health. These findings align with Farhan et al. [44], who emphasized the importance of bacterial strains capable of functioning under variable pH conditions, as they are more likely to succeed in biodegradation efforts, especially in the face of rapidly changing environmental conditions. The adaptability displayed by *Brevundimonas diminuta CB21* can significantly contribute to the success of contaminant degradation, making it a crucial factor to consider when selecting strains for bioremediation endeavors [26, 44, 49]. By understanding the significance of such adaptable bacteria, we can better design targeted and resilient bioremediation strategies to combat the persistent threat of pesticide pollution and safeguard both ecosystems and human well-being.

Most strains had optimum chlorpyrifos growth at an incubation temperature of 25°C except for MW2 (*Bacillus weihenstephanensis FB25M*), which recorded optimum growth at an incubation temperature of 30°C. In terms of degradation, majority of the isolates exhibited optimum degradation at 25°C, except for *Bacillus weihenstephanensis*, which had optimum degradation at 30°C. These findings differ slightly from previous studies that reported natural optimum temperature growth conditions at 37.5°C for *Alcaligenes faecalis* [50], 35°C for *Bacillus toyonensis* [40], 37°C for *Brevundimonas diminuta CB21* [41] and *Pseudomonas* spp. [51], and 30°C for *Bacillus weihenstephanensis* [52]. The variation could be due to the different environments in which the bacteria were grown, such as the MSM in the current study. Temperature has a huge impact on biological processes and can affect the growth of bacteria as well as influence bioremediation of pesticides [44, 53]. Mali et al. [43] demonstrated that the rate of degradation dropped from 99% at 32°C to less than 47% at 40°C and 58% at 22°C for *Bacillus* sp., which shows that optimum degradation takes place within a narrow range of temperature.

The results further revealed that temperature influences the extent of biodegradation of chlorpyrifos. The optimal temperature for bacteria degradation of chlorpyrifos in the current study was 25°C which falls within the range of normal soil temperature. These results corroborate with earlier studies that independently reported rapid biodegradation of chlorpyrifos at a temperature of between 20°C and 30°C [19, 54, 55]. Notably, at an increase in temperature to 37°C, the biodegrading of chlorpyrifos dropped. Naturally, metabolic rates are faster at elevated temperatures, irrespective of whether they are optimum. This could be due to the bacteria cell's highest biotic activity under these incubation conditions. The rate of biodegradation, however, can drop at higher temperatures since the important degradation enzymes are plasmid-borne and bacterial cells are known to lose plasmids at higher temperatures [56, 57].

The growth patterns of the bacterial isolates showed variations in response to different chlorpyrifos (CP) concentrations. Isolates MW1, MW3, MW4, MW5, MW6, and MW7 had higher optical densities (ODs) at a CP concentration of 25 mg/l on day 21, while isolate MW2 had a higher OD at 50 mg/l. Also, the degradation ability, as measured through the color intensity of formazan, was found to be optimum at a concentration of 25 mg/l, except for *Pseudomonas* sp. *strain PB845W*, *Brevundimonas diminuta*, and *uncultured bacterium clone 99*, whose optimum concentration was 100 mg/l. This observation is consistent with the previous findings by Iyer et al. [58] and Foong et al. [49], where different bacterial species, including *Bacillus* sp., *Pseudomonas* sp., *Achromobacter* sp., and *Ochrobactrum* sp., demonstrated CP degradation capabilities at concentrations of 100 mg/l within a range of 1 to 28 days. Statistics revealed a difference in ODs across isolates and incubation times that was statistically significant and that the interaction of key factors significantly affected ODs for all isolates. Additionally, the isolates' ODs at different incubation intervals showed a substantial difference. These results suggest that the concentration of CP present affects the growth patterns of various bacterial isolates and that their capacity to break down CP varies. At the high dose of 100 mg/l in the current investigation, growth was at its lowest. According to Sharma et al. [59], it is predicted that bacterial growth will be at its lowest at a high concentration of 100 mg/l of CP since high concentrations of CP can be toxic to bacterial cells and cause cell damage or death. The inhibition of crucial metabolic and enzyme activities may lead to a reduction in cell growth and reproduction. A loss of membrane integrity and a reduction in the capacity to absorb nutrients can arise from excessive concentrations of CP, which can also impair the cell membrane's ability to function correctly. As a result, the bacteria may be less able to thrive in the harsh environment and may be less able to break down CP at high concentrations [43]. However, several studies [49, 58] have reported the ability of various bacterial species including *Bacillus* sp., *Pseudomonas* sp., *Achromobacter* sp., and *Ochrobactrum* sp. to degrade CP at a concentration of 100 mg l^−1^ within a range of 1 to 28 days.

The observed differences in growth patterns and degradation ability of the bacterial isolates at various concentrations of CP indicate that each isolate possesses distinct abilities in the degradation of CP. There is observation that certain isolates exhibited increased optical densities (ODs) at a concentration of 25 mg/l of CP, while others demonstrated that higher ODs at 50 mg/l, underscore the significance of carefully selecting bacterial isolates to establish a consortium capable of effectively degrading CP. The results indicate that the interaction between the main factors has a notable impact on ODs, and there is a significant disparity in ODs among the isolates at different incubation intervals. These findings emphasize the importance of carefully selecting growth conditions for bacterial isolates in order to maximize their ability to degrade CP. In general, the findings of this study emphasize the significance of comprehending the growth patterns exhibited by various bacterial isolates in order to enhance their capacity for the biodegradation of CP [60, 61]. According to a study conducted by Sharma et al. [59], it has been observed that bacteria growth can be impeded by the presence of excessively elevated pesticide concentrations. The aforementioned critical observation underscores the importance of thoroughly evaluating the choice of bacterial isolates according to their growth reactions to specific CP concentrations, as this factor can have a substantial influence on their efficacy in bioremediation.

It is important to note that the optimal temperature and pH for chlorpyrifos degradation may also be influenced by other factors, such as the presence of other compounds or nutrients in the environment [62]. Further research is needed to fully understand the optimal conditions for chlorpyrifos degradation by different bacterial species. Significant differences in growth and biodegradation levels between the main factors and their interactions suggest that incubation period, parameters (pH, temperature, and concentration), and bacterium strain independently influence growth and chlorpyrifos biodegradation [63]. Regulating the studied parameters and incubation period may be more important in the biodegradation of chlorpyrifos. These findings can be linked to the evolution of different enzyme systems by microorganisms for the degradation of substrates and derived energy. Such enzymes have optimum values for pH and temperature at which they would have maximum activity. Thus, changes in environmental factors may affect enzyme activities [56]. This envisages interactions similar to those that may exist in nature and thus would help in better management of chlorpyrifos-contaminated ecosystems [21].

Furthermore, the findings of this study can inform that the formation of an effective consortium for degrading CP. *Alcaligenes* sp. has been previously reported to degrade chlorpyrifos through the hydrolysis of the P-O bond by using the metabolic pathway of chlorpyrifos hydrolase to break down the chlorpyrifos into 3,5,6-trichloro-2-pyridinol (TCP) and diethylthiophosphate (DETP), which are further broken down by other strains [43]. Also, *Pseudomonas* sp. and *Bacillus* spp. have been reported to degrade chlorpyrifos through oxidative and hydrolytic pathways [42, 64]. As a result, using a consortium of different strains can improve the efficiency of chlorpyrifos degradation by allowing different components of the compound to degrade, preventing the buildup of toxic intermediates, and speeding up the overall rate of degradation [21]. Multiple bacterial strain consortia have been shown to be more efficient at destroying organophosphorus pesticides than single strains in prior research [62, 65, 66]. The compatibility and synergistic abilities of the chosen strains, however, are critical to the consortium's success [26]. In order for the bacterial strains to develop and create enzymes that can break down chlorpyrifos, there may need to be an initial adaptation phase before the consortium can be established [43, 65]. Therefore, additional research is required to assess the interactions and connections between the consortium's chosen strains and to improve the conditions for chlorpyrifos breakdown. The consortia of bacteria's interactions and connections can significantly affect how pollutants like chlorpyrifos are degraded [26]. The bacterial strains chosen for the consortium should, in general, have complementary metabolic pathways, with each strain contributing to the breakdown of various parts of the target pollutant [21].

The findings of this study inform a recommendation for using its findings to enhance sustainable dairy production and prevent chlorpyrifos contamination. Bioremediation and ecosystem detoxification can use CP-degrading bacterial strains isolated herein. Dairy farmers can minimize chlorpyrifos in soil and water by using isolated bacterial strains. This improves milk quality, cattle health, and farm sustainability. The study's findings lay the groundwork for further research and practical chlorpyrifos contamination solutions in dairy production, enabling ecologically friendly practices and safer agricultural outputs.

The study has valuable insights but also limitations that warrant acknowledgment. Its focus is restricted to chlorpyrifos, excluding other pesticides, and is confined to laboratory settings, lacking field tests for real-world validation. Geographically, the research is limited to Nakuru County, Kenya, which may affect its generalizability. Additionally, the study falls short in examining the interactions among identified bacterial strains in a consortium, a key aspect for effective bioremediation. Future work should explore these consortium dynamics in complex settings and extend the research to diverse geographical areas for broader applicability in detoxifying CP-contaminated ecosystems.

## 5. Conclusion

This study has identified bacterial isolates with the potential to degrade chlorpyrifos (CP) and established their optimum growth and degradation conditions. The results showed that *Alcaligenes faecalis UWI9*, *Bacillus weihenstephanensis FB25M*, *Bacillus toyonensis 20SBZ2B*, *Alcaligenes* sp. *SCAU23*, *Pseudomonas* sp. *P_B845W*, *Brevundimonas diminuta CB21*, and *uncultured bacterium clone 99* can grow on and degrade CP. Most isolates achieved optimum growth at pH 7 and the rest at pH 5. The optimum growth and degradation temperature were determined to be 25°C, although there was general good growth across all the three temperatures. Majority of the isolates had higher growth and degradation at concentration of 25 mg/l, and one achieved maximum growth at 50 mg/l, while three showed maximum degradation at 100 mg/l. The data may provide a good basis for further research on the consortium reconstitution. The isolated bacteria strains in this study can be further developed into a consortium, based on the optimum conditions identified herein, to degrade CP in the laboratory and under field conditions. Besides, future studies should aim at coming up with the bacterial consortium for the best biodegradation of CP in different environmental conditions. With the array of the findings obtained herein, a further study has been commenced to utilize the synergetic interactions of the isolated bacteria in a consortium that will be used to develop a bioreactor for *in situ* bioremediation of CP-contaminated environments.

## Figures and Tables

**Table 1:** Soil physicochemical properties.

Property	Sampling site		
	Subukia	Nakuru Central	Molo
Soil pH	Medium (8.18)	Near neutral (6.92)	Near neutral (6.95)
Total nitrogen (%)	Adequate (0.46)	Adequate (0.37)	Adequate (0.37)
Total org. carbon (%)	Adequate (4.94)	Adequate (3.94)	Adequate (3.97)
Phosphorus (ppm)	High (104)	High (145)	High (90)
Potassium (meq%)	High (12.8)	High (10.8)	High (10.0)
Calcium (meq%)	High (16.0)	High (17.2)	High (19.0)
Magnesium (meq%)	High (6.78)	High (5.82)	High (5.78)
Manganese (meq%)	Adequate (1.15)	Adequate (1.43)	Adequate (1.63)
Copper (ppm)	Low (0.10)	Low (0.12)	Adequate (3.25)
Iron (ppm)	Adequate (12.2)	Adequate (83.7)	Adequate (248)
Zinc (ppm)	Adequate (81.8)	Adequate (79.8)	Adequate (96.2)
Sodium (meq%)	Adequate (0.64)	Adequate (1.06)	Adequate (1.02)
Inferred texture	Loamy Clay	Loamy Clay	Loamy Clay

**Table 2:** Summary of optimum conditions for growth and degradation of the bacterial strains.

Code	Isolate name	Growth optimum pH	Growth optimum temperature	Growth optimum concentration	Degradation optimum pH	Degradation optimum temperature	Degradation optimum concentration
MW1	*Alcaligenes faecalis*	7	25°C	25 mg/l	7	25°C	25 mg/l
MW2	*Bacillus weihenstephanensis*	7	30°C	50 mg/l	5	30°C	25 mg/l
MW3	*Bacillus toyonensis*	7	25°C	25 mg/l	5	25°C	25 mg/l
MW4	*Alcaligenes* sp. *strain SCAU23*	5	25°C	25 mg/l	5	25°C	25 mg/l
MW5	*Pseudomonas* sp. *strain PB845W*	7	25°C	25 mg/l	5	25°C	100 mg/l
MW6	*Brevundimonas diminuta*	7	25°C	25 mg/l	7	25°C	100 mg/l
MW7	*Uncultured bacterium clone 99*	5	25°C	25 mg/l	5	25°C	100 mg/l

## Data Availability

The gene sequences of the isolated bacteria were deposited in the NCBI GenBank. The link to the accession numbers is as follows: https://www.ncbi.nlm.nih.gov/nuccore/?term=Wepukhulu.

## Supplementary Materials

Supplementary Materials Tables with data on optical density for bacterial growth and CP degradation have been submitted as supplementary material (Tables S1, S2, and S3).
