# Supplementary material for: Optimization of Growth Conditions for Chlorpyrifos-Degrading Bacteria in Farm Soils in Nakuru County, Kenya
Source: Biomed Res Int. 2024 Jan 25;2024:1611871. doi: 10.1155/2024/1611871 (PMC10834098; doi:10.1155/2024/1611871)
Supplement: Supplementary Materials — Tables with data on optical density for bacterial growth and CP degradation have been submitted as supplementary material (Tables S1, S2, and S3). [file 1611871.f1.docx]

**Optimization of Growth Conditions for Chlorpyrifos Degrading Bacteria in Farm Soils in Nakuru County, Kenya**

**Authors:** MIRIAM WEPUKHULU^12#^, PETER WACHIRA^1^, NDERITU HURIA^1^, PAUL SIFUNA^3^, SULIMAN ESSUMAN^3^, MICAH ASAMBA^4^

**Supplementary material**

**Supplementary table S1: Effect of pH on growth of bacteria and CP degradation**

Note: Values are presented as $\bar{x}\pm SEM$ for three replicates. Means with dissimilar superscript lower-case alphabets within the same row are significantly different (P<0.05; One-Way ANOVA with Turkey’s *post hoc* test); Whereas means with dissimilar subscript uppercase alphabets within the same isolate and day are significantly different (P<0.05; paired student t-test statistic).

| **DAYS** | **λ (nm)** | **ISOLATES** | | | | | | | | | | | | | | | | | | | | |
| --- | --- | --- | --- | --- | --- | --- | --- | --- | --- | --- | --- | --- | --- | --- | --- | --- | --- | --- | --- | --- | --- | --- |
|  |  | **PH5MW1** | **PH 5MW2** | **PH 5MW3** | **PH 5MW4** | **PH 5MW5** | **PH 5MW6** | **PH 5MW7** | **PH 7MW1** | **PH 7MW2** | **PH 7MW3** | **PH 7MW4** | **PH 7MW5** | **PH 7MW6** | **PH 7MW7** | **PH 9MW1** | **PH 9MW2** | **PH 9MW3** | **PH 9MW4** | **PH 9MW5** | **PH 9MW6** | **PH9MW7** |
| **DAY 1** | **600** | 0.0943±0.00277^bcde^_A_ | 0.0951±0.00751^bcd^_A_ | 0.0814±0.000404^cdef^_A_ | 0.1175±0.0037^abc^_A_ | 0.0708±0.00294^cdefg^_A_ | 0.08495±0.00118^cdef^_A_ | 0.0812±0.00404^cdef^_A_ | 0.0617±0.0186^defg^_A_ | 0.11805±0.000144^abc^_A_ | 0.14555±0.00476^ab^_A_ | 0.1384±0.0322^ab^_A_ | 0.07855±0.00101^cdef^_A_ | 0.1511±0.000346^a^_A_ | 0.0334±0.000577^fg^_A_ | 0.11585±0.00557^abc^_B_ | 0.0834±0.00167^cdef^_A_ | 0.04735±0.00419^defg^_A_ | 0.0391±0.019^fg^_A_ | 0.04275±0.00303^efg^_A_ | 0.0789±0.00404^cdef^_A_ | 0.02125±0.00101^g^_A_ |
|  | **480** | 0.00265±0.00026^b^_B_ | 0.0176±0.000115^b^_B_ | 0.0185±0.000635^b^_B_ | 0.0224±0.00162^b^_B_ | 0.0204±0.00121^b^_B_ | 0.01055±0.00217^b^_B_ | 0.01445±0.00118^b^_B_ | 0.0009±0.000115^b^_B_ | 0.0115±0.00393^b^_B_ | 0.00675±0.00113^b^_B_ | 0.011±0.00052^b^_B_ | 0.0127±0.000808^b^_B_ | 0.01175±0.00188^b^_B_ | 0.0223±0.00699^b^_B_ | 0.1487±0.0712^a^_A_ | 0.00905±0.000375^b^_B_ | 0.0126±0.00606^b^_B_ | 0.00455±0.00026^b^_B_ | 0.00315±0.000722^b^_B_ | 0.00085±0.000087^b^_B_ | 0.00295±0.00107^b^_B_ |
| **DAY 5** | **600** | 0.3998±0.0475^a^_A_ | 0.351±0.00606^abc^_A_ | 0.3206±0.0444^abcde^_A_ | 0.334±0.00115^abcd^_A_ | 0.3456±0.00808^abc^_B_ | 0.3578±0.031^ab^_A_ | 0.3004±0.041^abcde^_A_ | 0.2369±0.00693^bcdef^_A_ | 0.2138±0.0401^cdef^_A_ | 0.1964±0.00831^ef^_A_ | 0.22±0.0136^cdef^_A_ | 0.24735±0.00436^bcdef^_A_ | 0.3047±0.0394^abcde^_A_ | 0.2407±0.0246^bcdef^_A_ | 0.1968±0.00895^def^_A_ | 0.224±0.013^bcdef^_A_ | 0.20025±0.00667^def^_A_ | 0.1938±0.00133^ef^_A_ | 0.16085±0.00696^f^_A_ | 0.2077±0.0378^def^_A_ | 0.2061±0.0291^def^_A_ |
|  | **480** | 0.2123±0.0163^b^_B_ | 0.21903±0.00852^b^_B_ | 0.18378±0.00797^b^_B_ | 0.18348±0.00849^b^_B_ | 0.597±0.232^a^_A_ | 0.18553±0.00153^b^_B_ | 0.16658±0.00242^b^_B_ | 0.10015±0.00476^b^_B_ | 0.0845±0.00052^b^_B_ | 0.07815±0.00159^b^_B_ | 0.07715±0.000433^b^_B_ | 0.0804±0.00289^b^_B_ | 0.0862±0.0112^b^_B_ | 0.056155±0.000436^b^_B_ | 0.11912±0.00268^b^_B_ | 0.14471±0.000557^b^_B_ | 0.13488±0.00473^b^_B_ | 0.13778±0.00283^b^_B_ | 0.13753±0.00274^b^_B_ | 0.13303±0.00447^b^_B_ | 0.11598±0.00266^b^_B_ |
| **DAY 9** | **600** | 0.562±0.0336^ab^_A_ | 0.5616±0.00953^ab^_A_ | 0.5329±0.0141^abc^_A_ | 0.43195±0.000029^abcdef^_A_ | 0.4787±0.0304^abcd^_A_ | 0.5943±0.0626^a^_A_ | 0.4739±0.026^abcd^_A_ | 0.372±0.0221^bcdef^_A_ | 0.25625±0.00707^ef^_A_ | 0.2506±0.00826^ef^_A_ | 0.29805±0.00621^def^_A_ | 0.5579±0.0292^ab^_A_ | 0.2408±0.0254^f^_A_ | 0.3309±0.0153^cdef^_A_ | 0.323±0.0246^def^_A_ | 0.3098±0.00485^def^_A_ | 0.2487±0.00797^ef^_A_ | 0.25055±0.000491^ef^_A_ | 0.448±0.14^abcde^_A_ | 0.27655±0.008^def^_A_ | 0.2361±0.0107^f^_A_ |
|  | **480** | 0.17915±0.00234^b^_B_ | 0.1549±0.00208^bc^_B_ | 0.1422±0.0165^bcd^_B_ | 0.9025±0.0612^a^_B_ | 0.1202±0.00964^bcdef^_B_ | 0.1281±0.0127^bcde^_B_ | 0.1265±0.014^bcdef^_B_ | 0.1179±0.0115^bcdef^_B_ | 0.1228±0.00231^bcdef^_B_ | 0.10745±0.000318^bcdef^_B_ | 0.1053±0.00127^bcdef^_B_ | 0.1078±0.0154^bcdef^_B_ | 0.0677±0.0159^def^_B_ | 0.0913±0.00266^cdef^_B_ | 0.08220±0.015^cdef^_B_ | 0.044100.00231^f^_B_ | 0.064100.0062^def^_B_ | 0.0670±0.015^def^_B_ | 0.10040^bcdef^_B_ | 0.042800.008^f^_B_ | 0.048300.002^ef^_B_ |
| **DAY 13** | **600** | 0.5927±0.0139^a^_A_ | 0.57665±0.00909^ab^_A_ | 0.5544±0.0241^ab^_A_ | 0.4879±0.00508^bc^_A_ | 0.49145±0.00938^ab^_A_ | 0.505±0.0116^ab^_A_ | 0.4891±0.0423^bc^_A_ | 0.3859±0.0187^cd^_A_ | 0.31055±0.00447^defgh^_A_ | 0.3449±0.00652^def^_A_ | 0.332±0.00121^defg^_A_ | 0.377±0.0428^d^_A_ | 0.2161±0.0214^h^_A_ | 0.3219±0.00427^defg^_A_ | 0.38085±0.0073^d^_A_ | 0.3499±0.00595^de^_A_ | 0.3253±0.0097^defg^_A_ | 0.23635±0.00932^gh^_A_ | 0.2445±0.00889^fgh^_A_ | 0.3227±0.0135^defg^_A_ | 0.2611±0.04^efgh^_A_ |
|  | **480** | 0.2313±0.0163^ab^_B_ | 0.2045±0.0166^abcd^_B_ | 0.2166±0.00964^abc^_B_ | 0.1891±0.00167^abcd^_B_ | 0.1798±0.01^abcd^_B_ | 0.1825±0.0167^abcd^_B_ | 0.1844±0.0193^abcd^_B_ | 0.1289±0.0259^bcd^_B_ | 0.18475±0.00251^abcd^_B_ | 0.1802±0.00473^abcd^_B_ | 0.2075±0.00167^abcd^_B_ | 0.1625±0.0231^abcd^_B_ | 0.16785±0.00228^abcd^_A_ | 0.18135±0.0032^abcd^_B_ | 0.09515±0.0039^cd^_B_ | 0.1183±0.0106^bcd^_B_ | 0.1101±0.0102^bcd^_B_ | 0.08525±0.00788^d^_B_ | 0.0822±0.00618^d^_B_ | 0.2783±0.0962^a^_B_ | 0.0844±0.0162^d^_B_ |
| **DAY 17** | **600** | 0.5845±0.0505^a^_A_ | 0.503±0.0109^ab^_A_ | 0.489±0.00981^ab^_A_ | 0.4212±0.0105^bcde^_A_ | 0.4369±0.0152^abcd^_A_ | 0.4456±0.0349^abcd^_A_ | 0.4667±0.0429^abc^_A_ | 0.3225±0.0117^cdef^_A_ | 0.2489±0.00364^fgh^_A_ | 0.2552±0.00346^fgh^_A_ | 0.26345±0.000029^fgh^_A_ | 0.3524±0.0568^bcdef^_A_ | 0.1651±0.0255^gh^_A_ | 0.3013±0.00312^defg^_A_ | 0.2496±0.0747^fgh^_A_ | 0.1644±0.0026^gh^_A_ | 0.2774±0.0161^efgh^_A_ | 0.25175±0.00141^fgh^_A_ | 0.3079±0.0151^defg^_A_ | 0.2346±0.00548^fgh^_A_ | 0.1298±0.0224^h^_A_ |
|  | **480** | 0.3235±0.0149^a^_B_ | 0.3234±0.0314^a^_B_ | 0.3043±0.0156^ab^_B_ | 0.245±0.0136^b^_B_ | 0.249500.008^ab^_B_ | 0.156800.018^cde^_B_ | 0.2542±0.000231^ab^_B_ | 0.1136±0.0169^cdef^_B_ | 0.134±0.0166^cdef^_B_ | 0.157±0.00162^cde^_B_ | 0.16855±0.00257^c^_B_ | 0.1611±0.0282^cd^_B_ | 0.08345±0.00534^ef^_B_ | 0.12575±0.00147^cdef^_B_ | 0.0783±0.0137^f^_B_ | 0.06835±0.00361^f^_B_ | 0.0883±0.0166^def^_B_ | 0.12065±0.00517^cdef^_B_ | 0.1061±0.013^cdef^_B_ | 0.11905±0.00956^cdef^_B_ | 0.0766±0.0163^f^_B_ |
| **DAY 21** | **600** | 0.6647±0.0254^ab^_A_ | 0.62395±0.00494^ab^_A_ | 0.59625±0.00892^b^_A_ | 0.51815±0.00586^bc^_A_ | 0.5366±0.0216^bc^_A_ | 0.535±0.028^bc^_A_ | 0.7716±0.0428^a^_A_ | 0.3264±0.0398^def^_A_ | 0.2938±0.00358^def^_A_ | 0.28915±0.0088^def^_A_ | 0.34625±0.00713^def^_A_ | 0.4269±0.0939^cd^_A_ | 0.2078±0.0203^f^_A_ | 0.27425±0.00257^def^_A_ | 0.3462±0.00387^def^_A_ | 0.3922±0.00237^cde^_A_ | 0.32415±0.00898^def^_A_ | 0.2145±0.00335^f^_A_ | 0.21005±0.00788^f^_A_ | 0.2543±0.0604^ef^_A_ | 0.2141±0.0348^f^_A_ |
|  | **480** | 0.2834±0.00999^b^_B_ | 0.5498940.008^a^_B_ | 0.2658±0.0229^b^_B_ | 0.2131±0.0122^b^_B_ | 0.2424±0.0141^b^_B_ | 0.22095±0.0043^b^_B_ | 0.2273±0.0316^b^_B_ | 0.1414±0.0192^b^_B_ | 0.1336±0.00589^b^_B_ | 0.1553±0.0105^b^_B_ | 0.17955±0.000664^b^_B_ | 0.1954±0.0323^b^_B_ | 0.0943±0.0135^b^_B_ | 0.1525±0.0132^b^_B_ | 0.1496±0.00548^b^_B_ | 0.1264±0.00704^b^_B_ | 0.10445±0.00684^b^_B_ | 0.089±0.00647^b^_B_ | 0.0801±0.00491^b^_B_ | 0.1177±0.0374^b^_B_ | 0.0808±0.0105^b^_B_ |

**Supplementary table S2: Effect of Temperature on growth of bacteria and CP degradation**

Note: Values are presented as $\bar{x}\pm SEM$ for three replicates. Means with dissimilar superscript lower-case alphabets within the same row are significantly different (P<0.05; One-Way ANOVA with Turkey’s *post hoc* test); Whereas means with dissimilar subscript uppercase alphabets within the same isolate and day are significantly different (P<0.05; paired student t-test statistic).

| **DAYS** | **λ (nm)** | **ISOLATES** | | | | | | | | | | | | | | | | | | | | |
| --- | --- | --- | --- | --- | --- | --- | --- | --- | --- | --- | --- | --- | --- | --- | --- | --- | --- | --- | --- | --- | --- | --- |
|  |  | **25°CMW1** | **25°C MW2** | **25°C MW3** | **25°C MW4** | **25°C MW5** | **25°C MW6** | **25°C MW7** | **30°C MW1** | **30°C MW2** | **30°C MW3** | **30°C MW4** | **30°C MW5** | **30°C MW6** | **30°C MW7** | **37°C MW1** | **37°C MW2** | **37°C MW3** | **37°C MW4** | **37°C MW5** | **37°C MW6** | **37°C MW7** |
| **DAY 1** | **600** | 0.0402±0.00641^bc^_A_ | 0.0917±0.00606^bc­^_A_ | 0.1108±0.046^bc^_A_ | 0.1512±0.0417^abc^_A_ | 0.38±0.18^a^_A_ | 0.1146±0.00693^bc^_A_ | 0.05205±0.00199^bc^_A_ | 0.0617±0.0186^bc^_A_ | 0.11805±0.000144^bc^_A_ | 0.14555±0.00476^bc^_A_ | 0.1384±0.0322^bc^_A_ | 0.07855±0.00101^bc^_A_ | 0.1511±0.000346^abc^_A_ | 0.0334±0.000577^c^_A_ | 0.2708±0.0329^ab^_A_ | 0.1372±0.00219^bc^_A_ | 0.1399±0.0208^bc^_A_ | 0.1491±0.0106^abc^_A_ | 0.18175±0.0088^abc^_A_ | 0.14615±0.00315^abc^_A_ | 0.125±0.0131^bc^_A_ |
|  | **480** | 0.02185±0.000029^bcd^_B_ | 0.0192±0.00329^bcde^_B_ | 0.0092±0.00185^cdef^_B_ | 0.0423±0.003^a^_B_ | 0.0394±0.000808^a^_B_ | 0.0195±0.000693^bcde^_B_ | 0.0088±0.00185^def^_B_ | 0.0009±0.000115^f^_B_ | 0.0115±0.00393^bcdef^_B_ | 0.00675±0.00113^ef^_A_ | 0.011±0.00052^bcdef^_B_ | 0.0127±0.000808^bcdef^_B_ | 0.01175±0.00188^bcdef^_B_ | 0.0223±0.00699^bcd^_B_ | 0.04755±0.00286^a^_B_ | 0.02105±0.00263^bcd^_B_ | 0.01905±0.00113^bcde^_B_ | 0.0214±0.00352^bcd^_B_ | 0.01825±0.00182^bcde^_B_ | 0.02285±0.00211^bc^_B_ | 0.0242±0.00121^b^_B_ |
| **DAY 5** | **600** | 0.1792±0.0477^a^_A_ | 0.15605±0.00828^a^_A_ | 0.2284±0.0349^a^_A_ | 0.2955±0.0294^a^_A_ | 0.2287±0.00548^a^_A_ | 0.17645±0.00927^a^_A_ | 0.24305±0.00113^a^_A_ | 0.2369±0.00693^a^_A_ | 0.2138±0.0401^a^_A_ | 0.1964±0.00831^a^_A_ | 0.22±0.0136^a^_A_ | 0.24735±0.00436^a^_A_ | 0.3047±0.0394^a^_A_ | 0.2407±0.0246^a^_A_ | 0.2211±0.0376^a^_A_ | 0.185±0.0143^a^_A_ | 0.1832±0.00762^a^_A_ | 0.2551±0.0664^a^_A_ | 0.19935±0.00124^a^_A_ | 0.2506±0.0589^a^_A_ | 0.2324±0.0225^a^_A_ |
|  | **480** | 0.0647±0.00306^bcd^_B_ | 0.0618±0.00052^bcde^_B_ | 0.07585±0.0032^abc^_B_ | 0.0852±0.000115^abc^_B_ | 0.0821±0.0041^abc^_B_ | 0.0669±0.00185^bcd^_B_ | 0.0587±0.000173^bcde^_B_ | 0.10015±0.00476^a^_B_ | 0.0845±0.00052^abc^_B_ | 0.07815±0.00159^abc^_B_ | 0.07715±0.000433^abc^_B_ | 0.0804±0.00289^abc^_B_ | 0.0862±0.0112^ab^_B_ | 0.056155±0.000436^cde^_B_ | 0.04555±0.00263^def^_B_ | 0.0221±0.00456^f^_B_ | 0.0664±0.00346^bcd^_B_ | 0.0582±0.00231^bcde^_B_ | 0.0414±0.0182^def^_B_ | 0.033±0.00127^ef^_B_ | 0.04405±0.00817^def^_B_ |
| **DAY 9** | **600** | 0.30895±0.00263^bcdef^_A_ | 0.25775±0.00245^cdef^_A_ | 0.2555±0.026^cdef^_A_ | 0.31875±0.00499^bcde^_A_ | 0.2903±0.0129^bcdef^_A_ | 0.2984±0.00329^bcdef^_A_ | 0.23028±0.00299^ef^_A_ | 0.372±0.0221^b^_A_ | 0.25625±0.00707^cdef^_A_ | 0.2506±0.00826^cdef^_A_ | 0.29805±0.00621^bcdef^_A_ | 0.5579±0.0292^a^_A_ | 0.2408±0.0254^def^_A_ | 0.3309±0.0153^bcd^_A_ | 0.603±0.0441^a^_A_ | 0.339±0.0286^bc^_A_ | 0.2584±0.00543^cdef^_A_ | 0.2441±0.0221^cdef^_A_ | 0.30915±0.0017^bcdef^_A_ | 0.2164±0.00115^f^_A_ | 0.22675±0.00817^ef^_A_ |
|  | **480** | 0.0883±0.0162^bcde^_B_ | 0.1222±0.00387^ab^_B_ | 0.12±0.00658^ab^_B_ | 0.1453±0.00242^a^_B_ | 0.1453±0.00242^a^_B_ | 0.1183±0.0176^ab^_B_ | 0.06535±0.00517^de^_B_ | 0.1179±0.0115^ab^_B_ | 0.1228±0.00231^ab^_B_ | 0.10745±0.000318^abcd^_B_ | 0.1053±0.00127^abcd^_B_ | 0.1078±0.0154^abcd^_B_ | 0.0677±0.0159^cde^_B_ | 0.0913±0.00266^bcde^_B_ | 0.112600.008^ab^_B_ | 0.05170±0.008^e^_B_ | 0.09110±0.0145^bcde^_B_ | 0.08210±0.0115^bcde^_B_ | 0.11460±0.0105^ab^_B_ | 0.10890±0.0015^abc^_B_ | 0.1085±0.00231^abc^_B_ |
| **DAY 13** | **600** | 0.3945±0.0566^bcde^_A_ | 0.3851±0.0648^bcde^_A_ | 0.4089±0.0588^bcde^_A_ | 0.5711±0.0124^a^_A_ | 0.4662±0.0514^abc^_A_ | 0.5007±0.00572^ab^_A_ | 0.50315±0.000202^ab^_A_ | 0.3859±0.0187^bcde^_A_ | 0.31055±0.00447^cdefg^_A_ | 0.3449±0.00652^bcdefg^_A_ | 0.332±0.00121^cdefg^_A_ | 0.377±0.0428^bcdef^_A_ | 0.2161±0.0214^fg^_A_ | 0.3219±0.00427^cdefg^_A_ | 0.4383±0.0167^abcd^_A_ | 0.213±0.0344^g^_A_ | 0.2842±0.0164^defg^_A_ | 0.3045±0.0243^defg^_A_ | 0.36275±0.00395^bcdefg^_A_ | 0.39275±0.00286^bcde^_A_ | 0.25385±0.00632^efg^_A_ |
|  | **480** | 0.1105±0.0171^bcdef^_B_ | 0.11105±0.00026^bcdef^_B_ | 0.1017±0.011^cdef^_B_ | 0.1185±0.00133^bcdef^_B_ | 0.1179±0.0146^bcdef^_B_ | 0.0845±0.00751^ef^_B_ | 0.1456±0.000173^abcdef^_B_ | 0.1289±0.0259^abcdef^_B_ | 0.18475±0.00251^ab^_B_ | 0.1802±0.00473^abc^_B_ | 0.2075±0.00167^a^_B_ | 0.1625±0.0231^abcde^_B_ | 0.16785±0.00228^abcd^_B_ | 0.18135±0.0032^abc^_B_ | 0.1383±0.013^abcdef^_B_ | 0.0826±0.0177^ef^_B_ | 0.10795±0.0013^bcdef^_B_ | 0.1082±0.0157^bcdef^_B_ | 0.11605±0.00707^bcdef^_B_ | 0.0897±0.0349^def^_B_ | 0.0796±0.0276^f^_B_ |
| **DAY 17** | **600** | 0.2844±0.0713^abc^_A_ | 0.32535±0.000779^abc^_A_ | 0.2975±0.0277^abc^_A_ | 0.42915±0.00176^a^_A_ | 0.219±0.107^bc^_A_ | 0.3505±0.00318^abc^_A_ | 0.3593±0.0227^abc^_A_ | 0.3225±0.0117^abc^_A_ | 0.2489±0.00364^abc^_A_ | 0.2552±0.00346^abc^_A_ | 0.26345±0.000029^abc^_A_ | 0.3524±0.0568^abc^_A_ | 0.1651±0.0255^c^_A_ | 0.3013±0.00312^abc^_A_ | 0.29995±0.00979^abc^_A_ | 0.1757±0.0273^c^_A_ | 0.4173±0.000808^ab^_A_ | 0.2228±0.0168^bc^_A_ | 0.2701±0.00589^abc^_A_ | 0.3321±0.000693^abc^_A_ | 0.3025±0.0752^abc^_A_ |
|  | **480** | 0.1356±0.0357^a^_B_ | 0.0996±0.0359^a^_B_ | 0.1628±0.00889^a^_B_ | 0.10675±0.00251^a^_B_ | 0.1686±0.00502^a^_B_ | 0.1013±0.0378^a^_B_ | 0.1655±0.0156^a^_B_ | 0.1136±0.0169^a^_B_ | 0.134±0.0166^a^_B_ | 0.157±0.00162^a^_B_ | 0.16855±0.00257^a^_B_ | 0.1611±0.0282^a^_B_ | 0.08345±0.00534^a^_B_ | 0.12575±0.00147^a^_B_ | 0.12975±0.00644^a^_B_ | 0.0802±0.0136^a^_B_ | 0.0982±0.00733^a^_B_ | 0.1042±0.0108^a^_B_ | 0.11505±0.00245^a^_B_ | 0.12645±0.00286^a^_B_ | 0.08805±0.00268^a^_B_ |
| **DAY 21** | **600** | 0.4734±0.0813^abc^_A_ | 0.47795±0.00759^ab^_A_ | 0.4304±0.0489^abcdef^_A_ | 0.53325±0.00921^a^_A_ | 0.4685±0.0277^abcd^_A_ | 0.5301±0.00404^a^_A_ | 0.4579±0.0233^abcde^_A_ | 0.3264±0.0398^bcdefg^_A_ | 0.2938±0.00358^cdefg^_A_ | 0.28915±0.0088^defg^_A_ | 0.34625±0.00713^bcdefg^_A_ | 0.4269±0.0939^abcdef^_A_ | 0.2078±0.0203^g^_A_ | 0.27425±0.00257^fg^_A_ | 0.46555±0.00222^abcd^_A_ | 0.2581±0.0421^fg^_A_ | 0.2974±0.0244^bcdefg^_A_ | 0.3419±0.024^bcdefg^_A_ | 0.35935±0.00136^abcdefg^_A_ | 0.37665±0.00494^abcdefg^_A_ | 0.2788±0.000924^efg^_A_ |
|  | **480** | 0.162±0.0397^abcd^_B_ | 0.1589±0.00595^abcd^_B_ | 0.2075±0.014^abc^_B_ | 0.2111±0.00843^ab^_B_ | 0.2308±0.0139^a^_B_ | 0.1739±0.0167^abcd^_B_ | 0.127±0.0593^abcd^_B_ | 0.1414±0.0192^abcd^_B_ | 0.1336±0.00589^abcd^_B_ | 0.1553±0.0105^abcd^_B_ | 0.17955±0.000664^abcd^_B_ | 0.1954±0.0323^abcd^_B_ | 0.0943±0.0135^d^_B_ | 0.1525±0.0132^abcd^_B_ | 0.16295±0.00921^abcd^_B_ | 0.0877±0.0166^d^_B_ | 0.1262±0.0132^abcd^_B_ | 0.1403±0.017^abcd^_B_ | 0.1313±0.00843^abcd^_B_ | 0.103±0.00375^bcd^_B_ | 0.0996±0.01^cd^_B_ |

**Supplementary Table S3: Concentration**

Note: Values are presented as $\bar{x}\pm SEM$ for three replicates. Means with dissimilar superscript lower-case alphabets within the same row are significantly different (P<0.05; One-Way ANOVA with Turkey’s *post hoc* test); Whereas means with dissimilar subscript uppercase alphabets within the same isolate and day are significantly different (P<0.05; paired student t-test statistic).

| **DAYS** | **λ** | **ISOLATES** | | | | | | | | | | | | | | | | | | | | |
| --- | --- | --- | --- | --- | --- | --- | --- | --- | --- | --- | --- | --- | --- | --- | --- | --- | --- | --- | --- | --- | --- | --- |
|  |  | **25ppmMW1** | **25ppmMW2** | **25ppmMW3** | **25ppmMW4** | **25ppmMW5** | **25ppmMW6** | **25ppmMW7** | **50ppmMW1** | **50ppmMW2** | **50ppmMW3** | **50ppmMW4** | **50ppmMW5** | **50ppmMW6** | **50ppmMW7** | **100ppmMW1** | **100ppmMW2** | **100ppmMW3** | **100ppmMW4** | **100ppmMW5** | **100ppmMW6** | **100ppmMW7** |
| **DAY 1** | **λ=600 nm** | 0.19095±0.00401^abcd^_A_ | 0.1954±0.00196^abcd^_A_ | 0.1867±0.00479^bcd^_A_ | 0.1476±0.00277^def^_A_ | 0.1558±0.00312^cdef^_A_ | 0.17525±0.00701^cde^_A_ | 0.10825±0.000664^fgh^_A_ | 0.1615±0.00958^cdef^_A_ | 0.2503±0.0135^a^_A_ | 0.2161±0.0221^abc^_A_ | 0.1416±0.0101^def^_A_ | 0.24255±0.0095^ab^_A_ | 0.1618±0.0148^cdef^_A_ | 0.1394±0.0111^defg^_A_ | 0.0617±0.0186^hi^_A_ | 0.11805±0.000144^efgh^_A_ | 0.14555±0.00476^def^_A_ | 0.1384±0.0322^defg^_A_ | 0.07855±0.00101^ghi^_A_ | 0.1511±0.000346^def^_A_ | 0.0334±0.000577^i^_A_ |
|  | **λ=480 nm** | 0.09095±0.00401^a^_A_ | 0.0953±0.00191^a^_B_ | 0.0882±0.00566^ab^_B_ | 0.0476±0.00277^de^_A_ | 0.0558±0.00312^cd^_B_ | 0.07225±0.00528^bc^_B_ | 0.07005±0.000779^c^_B_ | 0.00845±0.00268^ij^_B_ | 0.0071±0.00208^ij^_A_ | 0.0429±0.00473^def^_B_ | 0.03485±0.0024^efg^_B_ | 0.03735±0.00309^efg^_B_ | 0.02945±0.00141^fgh^_B_ | 0.00425±0.000606^j^_B_ | 0.0009±0.000115^j^_A_ | 0.0115±0.00393^ij^_B_ | 0.00675±0.00113^ij^_B_ | 0.011±0.00052^ij^_A_ | 0.0127±0.000808^hij^_B_ | 0.01175±0.00188^ij^_B_ | 0.0223±0.00699^ghi^_A_ |
| **DAY 5** | **λ=600 nm** | 0.1105±0.0129^e^_A_ | 0.1601±0.0298^cde^_A_ | 0.1563±0.0484^cde^_A_ | 0.2415±0.0108^bc^_A_ | 0.2326±0.0137^bcde^_A_ | 0.2104±0.0218^bcde^_A_ | 0.1733±0.0161^cde^_A_ | 0.3681±0.0424^a^_A_ | 0.1178±0.016^de^_A_ | 0.2015±0.0149^bcde^_A_ | 0.204±0.011^bcde^_A_ | 0.2375±0.00225^bcd^_A_ | 0.20795±0.00701^bcde^_A_ | 0.2441±0.000231^bc^_A_ | 0.2369±0.00693^bcd^_A_ | 0.2138±0.0401^bcde^_A_ | 0.1964±0.00831^bcde^_A_ | 0.22±0.0136^bcde^_A_ | 0.24735±0.00436^abc^_A_ | 0.3047±0.0394^ab^_A_ | 0.2407±0.0246^bcd^_A_ |
|  | **λ=480 nm** | 0.01565±0.00026^i^_B_ | 0.04525±0.0028^fgh^_B_ | 0.0242±0.00133^i^_B_ | 0.03375±0.000953^ghi^_B_ | 0.0481±0.00115^fg^_B_ | 0.02015±0.000144^i^_B_ | 0.0279±0.00519^hi^_B_ | 0.15635±0.000318^a^_B_ | 0.1124±0.00156^bc^_A_ | 0.127±0.0041^b^_B_ | 0.1178±0.00121^bc^_B_ | 0.12015±0.00367^b^_B_ | 0.11555±0.000491^bc^_B_ | 0.1217±0.00566^b^_B_ | 0.10015±0.00476^cd^_B_ | 0.0845±0.00052^de^_B_ | 0.07815±0.00159^e^_B_ | 0.07715±0.000433^e^_B_ | 0.0804±0.00289^e^_B_ | 0.0862±0.0112^de^_B_ | 0.056155±0.000436^f^_B_ |
| **DAY 9** | **λ=600 nm** | 0.34425±0.0039^defgh^_A_ | 0.3412±0.0178^defgh^_A_ | 0.4139±0.00883^cd^_A_ | 0.5131±0.0105^ab^_A_ | 0.29675±0.00234^ghij^_A_ | 0.30475±0.00436^fghij^_A_ | 0.285±0.00687^hijk^_A_ | 0.5001±0.0302^ab^_A_ | 0.21605±0.00471^k^_A_ | 0.4441±0.0162^bc^_A_ | 0.2864±0.0112^hijk^_A_ | 0.3967±0.00635^cde^_A_ | 0.376±0.00468^cdef^_A_ | 0.33395±0.00938^efghi^_A_ | 0.372±0.0221^cdefg^_A_ | 0.25625±0.00707^ijk^_A_ | 0.2506±0.00826^jk^_A_ | 0.29805±0.00621^fghij^_A_ | 0.5579±0.0292^a^_A_ | 0.2408±0.0254^jk^_A_ | 0.3309±0.0153^efghi^_A_ |
|  | **λ=480 nm** | 0.0841±0.00883^b^_B_ | 0.1112±0.0239^ab^_B_ | 0.07475±0.000722^b^_B_ | 0.10305±0.00834^ab^_B_ | 0.09115±0.00165^ab^_B_ | 0.1149±0.0326^ab^_B_ | 0.07645±0.00251^b^_B_ | 0.08080±0.00251^b^_B_ | 0.08970±0.00251^ab^_B_ | 0.0690±0.00251^b^_B_ | 0.07960±0.00251^b^_B_ | 0.12350±0.00251^ab^_B_ | 0.14590±0.00251^a^_B_ | 0.07380±0.00251^b^_B_ | 0.1179±0.0115^ab^_B_ | 0.1228±0.00231^ab^_B_ | 0.10745±0.000318^ab^_B_ | 0.1053±0.00127^ab^_B_ | 0.1078±0.0154^ab^_B_ | 0.0677±0.0159^b^_B_ | 0.0913±0.00266^ab^_B_ |
| **DAY 13** | **λ=600 nm** | 0.5533±0.0901^a^_A_ | 0.5401±0.0376^ab^_A_ | 0.4361±0.00537^abcdef^_A_ | 0.45195±0.0099^abcdef^_A_ | 0.4395±0.0119^abcdef^_A_ | 0.4098±0.015^bcdef^_A_ | 0.4154±0.012^abcdef^_A_ | 0.52585±0.00338^abc^_A_ | 0.4729±0.0266^abcde^_A_ | 0.5129±0.0125^abcd^_A_ | 0.4001±0.0128^bcdef^_A_ | 0.493±0.0237^abcd^_A_ | 0.4985±0.0174^abcd^_A_ | 0.429±0.00878^abcdef^_A_ | 0.3859±0.0187^cdef^_A_ | 0.31055±0.00447^fg^_A_ | 0.3449±0.00652^efg^_A_ | 0.332±0.00121^efg^_A_ | 0.377±0.0428^def^_A_ | 0.2161±0.0214^g^_A_ | 0.3219±0.00427^fg^_A_ |
|  | **λ=480 nm** | 0.152±0.0236^bcd^_B_ | 0.1774±0.0189^abcd^_B_ | 0.1514±0.00167^bcd^_B_ | 0.1799±0.00699^abcd^_B_ | 0.2049±0.00866^ab^_B_ | 0.2265±0.0117^a^_B_ | 0.2002±0.0112^abc^_B_ | 0.13935±0.00153^cd^_B_ | 0.16425±0.00915^abcd^_B_ | 0.17155±0.00395^abcd^_B_ | 0.13385±0.00471^d^_B_ | 0.18785±0.00915^abcd^_B_ | 0.185±0.0119^abcd^_B_ | 0.18485±0.000202^abcd^_B_ | 0.1289±0.0259^d^_B_ | 0.18475±0.00251^abcd^_B_ | 0.1802±0.00473^abcd^_B_ | 0.2075±0.00167^ab^_B_ | 0.1625±0.0231^bcd^_B_ | 0.16785±0.00228^abcd^_B_ | 0.18135±0.0032^abcd^_B_ |
| **DAY 17** | **λ=600 nm** | 0.4557±0.0231^abcd^_A_ | 0.4736±0.0223^abcd^_A_ | 0.4059±0.0162^abcdef^_A_ | 0.50525±0.00499^ab^_A_ | 0.397±0.00479^bcdef^_A_ | 0.4757±0.0121^abc^_A_ | 0.40705±0.00453^abcdef^_A_ | 0.4496±0.0292^abcde^_A_ | 0.373±0.0329^cdefgh^_A_ | 0.4595±0.0369^abcd^_A_ | 0.3797±0.0262^bcdefg^_A_ | 0.5328±0.014^a^_A_ | 0.4613±0.0457^abcd^_A_ | 0.3446±0.0185^defgh^_A_ | 0.3225±0.0117^efgh^_A_ | 0.2489±0.00364^hi^_A_ | 0.2552±0.00346^ghi^_A_ | 0.26345±0.000029^ghi^_A_ | 0.3524±0.0568^cdefgh^_A_ | 0.1651±0.0255^i^_A_ | 0.3013±0.00312^fgh^_A_ |
|  | **λ=480 nm** | 0.1815±0.0185^abcde^_B_ | 0.1976±0.0111^abcd^_B_ | 0.20105±0.0065^abcd^_B_ | 0.1919±0.00052^abcd^_B_ | 0.2468±0.00883^a^_B_ | 0.2344±0.015^ab^_B_ | 0.19775±0.00447^abcd^_B_ | 0.18245±0.00725^abcde^_B_ | 0.2578±0.0134^a^_B_ | 0.18225±0.00424^abcde^_B_ | 0.2091±0.0294^abc^_B_ | 0.1862±0.0176^abcde^_B_ | 0.1842±0.0187^abcde^_B_ | 0.1915±0.0186^abcd^_B_ | 0.1136±0.0169^ef^_B_ | 0.134±0.0166^cdef^_B_ | 0.157±0.00162^cdef^_B_ | 0.16855±0.00257^bcde^_B_ | 0.1611±0.0282^bcdeB^ | 0.08345±0.00534^f^_B_ | 0.12575±0.00147^def^_B_ |
| **DAY 21** | **λ=600 nm** | 0.5916±0.0978^ab^_A_ | 0.46665±0.0039^abcdef^_A_ | 0.5179±0.0815^abcde^_A_ | 0.4254±0.0194^bcdefg^_A_ | 0.4926±0.0334^abcdef^_A_ | 0.403±0.0111^bcdefg^_A_ | 0.38675±0.00026^bcdefg^_A_ | 0.6124±0.0501^ab^_A_ | 0.5763±0.0816^abc^_A_ | 0.5545±0.00121^abcd^_A_ | 0.49115±0.00188^abcdef^_A_ | 0.5981±0.041^ab^_A_ | 0.701±0.0256^a^_A_ | 0.4757±0.019^abcdef^_A_ | 0.3264±0.0398^defg^_A_ | 0.2938±0.00358^efg^_A_ | 0.28915±0.0088^efg^_A_ | 0.34625±0.00713^cdefg^_A_ | 0.4269±0.0939^bcdefg^_A_ | 0.2078±0.0203^g^_A_ | 0.27425±0.00257^fg^_A_ |
|  | **λ=480 nm** | 0.2127±0.0165^abcd^_B_ | 0.2036±0.00393^abcd^_B_ | 0.1731±0.014^abcde^_B_ | 0.2363±0.0329^ab^_B_ | 0.2141±0.00393^abcd^_B_ | 0.1892±0.00779^abcd^_B_ | 0.1758±0.0147^abcde^_B_ | 0.1937±0.00624^abcd^_B_ | 0.1783±0.0204^abcde^_B_ | 0.2464±0.0229^a^_B_ | 0.1951±0.00104^abcd^_B_ | 0.2264±0.0115^abc^_B_ | 0.2083±0.0346^abcd^_B_ | 0.2056±0.00352^abcd^_B_ | 0.1414±0.0192^cde^_B_ | 0.1336±0.00589^de^_B_ | 0.1553±0.0105^abcde^_B_ | 0.17955±0.000664^abcde^_B_ | 0.1954±0.0323^abcd^_B_ | 0.0943±0.0135^e^_B_ | 0.1525±0.0132^bcde^_B_ |
